# The detection of absence seizures using cross-frequency coupling analysis with a deep learning network

**DOI:** 10.21203/rs.3.rs-4178484/v1

**Published:** 2024-04-10

**Authors:** Andrei V. Medvedev, Bar Lehmann

**Affiliations:** Georgetown University Medical Center; Georgetown University Medical Center

**Keywords:** Absence seizure, Epilepsy, EEG, spectral analysis, cross-frequency coupling

## Abstract

High frequency oscillations are important novel biomarkers of epileptogenic tissue. The interaction of oscillations across different time scales is revealed as cross-frequency coupling (CFC) representing a high-order structure in the functional organization of brain rhythms. New artificial intelligence methods such as deep learning neural networks can provide powerful tools for automated analysis of EEG. Here we present a Stacked Sparse Autoencoder (SSAE) trained to recognize absence seizure activity based on the cross-frequency patterns within scalp EEG. We used EEG records from the Temple University Hospital database. Absence seizures (n = 94) from 12 patients were taken into analysis along with segments of background activity. Half of the records were selected randomly for network training and the second half were used for testing. Power-to-power coupling was calculated between all frequencies 2–120 Hz pairwise using the EEGLAB toolbox. The resulting CFC matrices were used as training or testing inputs to the autoencoder. The trained network was able to recognize background and seizure segments (not used in training) with a sensitivity of 96.3%, specificity of 99.8% and overall accuracy of 98.5%. Our results provide evidence that the SSAE neural networks can be used for automated detection of absence seizures within scalp EEG.

## Introduction

Brain oscillations span frequencies across a range of several orders of magnitude from the Berger bands below 30 Hz (delta, theta, alpha, beta) up to the high frequency bands of gamma, ripple, and fast ripple (30–600 Hz). Our study was inspired by emerging evidence that brain oscillations do not work independently from each other but interact in a very complex and well-coordinated way known as cross-frequency coupling (CFC). Cross-frequency coupling plays an important role in the functional organization of neural networks at different spatial and temporal scales. This coupling represents a high-order structure in the functional organization of brain rhythms and is likely to reflect different functional states of the brain.

In recent years, there has been a burgeoning interest in high-frequency oscillations (HFOs) driven by emerging evidence suggesting their involvement in cognitive functions^1–8^. Also, heightened activity in these frequency ranges has been observed in pathological conditions and, in particular, numerous studies have demonstrated a significant increase in HFOs in the context of epilepsy. Those studies have revealed that HFOs are one of the most common early manifestations recorded within minutes before seizure onset and appear to be a reliable EEG correlate of ictal onset zone^9–19^. Several research groups have suggested that detection of HFOs is necessary for a more accurate localization of epileptogenic tissue. Improvements in accuracy may improve surgical outcome in patients with localization-related intractable epilepsy because the removal of HFO-generating areas correlates with good surgical outcomes^13,14,18,20–28^. Thus, in addition to epileptic discharges, HFOs are now considered as an important biomarker of epileptogenic tissue.

High-frequency bursts are frequently accompanied by low-frequency waveforms, such as sharp waves and spikes. These patterns may signify specific forms of cross-frequency coupling. The most typical examples pertinent to epilepsy include the Ripple-on-Spike, where a high-frequency burst is riding on a spike, as well as the Ripple-on-Oscillation, where a high-frequency burst is riding on a slow wave. Given that epileptic seizures are often accompanied by specific patterns of cross-frequency coupling between slow and fast activity, we are exploring the possibility that cross-frequency coupling may be used as a tool for automated detection of seizures.

In this study we focus on absence seizures because they are the most common type of childhood epilepsy and represent several challenges to clinicians. These challenges stem from the unique characteristics of absence seizures and their impact on the individuals who experience them. Absence seizures are often subtle and brief, lasting only a few seconds. The lack of convulsions or dramatic physical movements can make them less noticeable to observers, including clinicians. This subtlety may lead to under-recognition and misinterpretation of the seizures. Furthermore, the presentation of absence seizures can vary among individuals. Some may experience typical absences with staring spells, while others may exhibit more atypical features, such as subtle facial movements or eye fluttering. This variability makes diagnosis and recognition challenging for clinicians. The symptoms of absence seizures can also overlap with other neurological or psychiatric conditions. Clinicians must differentiate absence seizures from conditions like daydreaming, attention-deficit/hyperactivity disorder (ADHD), or other types of seizures. This requires a comprehensive clinical evaluation, including EEG monitoring. Thus, while absence seizures are generally considered less severe than some other types of seizures, they present a range of challenges for clinicians, from the subtlety of their presentation to their potential impact on cognitive function and daily life. Accurate counting of absence seizures is crucial for optimizing therapy. Current diagnostics rely on clinical history, in-hospital video-EEG monitoring, and patient-maintained seizure diaries. However, research indicates that patients report only 6% of all experienced absences^29^, while caregivers report 14%^30^. Therefore, a multipronged approach, including careful clinical evaluation and long-term EEG monitoring, is essential to address those challenges and to provide optimal care for individuals with absence seizures.

Cutting-edge artificial intelligence techniques, particularly deep learning (DL) neural networks, offer robust tools for the automated analysis of EEG, including the exploration of cross-frequency coupling between distinct EEG rhythms. Our methodology leverages DL neural networks with the objective of identifying various cross-frequency patterns and employing them for the automated detection of seizures. In this context, we introduce a Stacked Sparse Autoencoder (SSAE) specifically trained to identify absence seizure activity based on unique cross-frequency coupling patterns within scalp EEG.

## Methods

We used scalp EEG records (sampling frequency = 256 Hz) from the open source Temple University Hospital database (the TUSZ corpus,^31^). This dataset contains de-identified segments of EEG from epilepsy patients of different ages with seizures annotated by neurologists. Informed consent was obtained from all subjects and/or their legal guardian(s) as appropriate for patient’s age. The study was carried out in accordance with the relevant ethical guidelines and regulations and was approved by the Georgetown-MedStar Institutional Review Board. The TUSZ EEG files contained either background activity (during the interictal periods) or seizures with immediately preceding preictal as well as postictal activity. The duration of EEG segments varied from a few seconds up to several minutes. The annotations for each EEG segment contained the seizure type (as determined both by EEG as well as clinical/behavioral characteristics) alongside the respective onset and offset times of that seizure. All absence seizures (n = 94) from 12 patients were taken into analysis. The same number of segments of background activity were also randomly selected from all available background segments so that they approximately matched seizure segments in duration.

Our preprocessing pipeline included the following steps: re-referencing to common average, notch filtering (line frequency and its harmonics using the CleanLine EEGLAB toolbox), Independent Component (IC) decomposition using the AMICA algorithm, and the automated removal of artifactual (‘bad’) ICs using the ICLabel algorithm^32^. An IC was removed based on the following two criteria. First, if any of its probabilities (assigned by the ICLabel algorithm as a percentage) of being ‘muscle’, ‘eye’, ‘heart’, ‘line noise’ or ‘channel noise’ was greater than the probability of being ‘brain’ or ‘other’. Second, if the sum of the percentages of all the above artifactual assignments for this IC was greater than 50%.

Of several available measures of cross-frequency coupling, we chose the power-to-power coupling. Power-to-power (or amplitude-to-amplitude) coupling is methodologically easier to measure because it is based on simple correlation between the time courses of power changes within specific frequency bands. Also, the metrics based on correlation of signal power/amplitude seem to be more robust against the effect of noise compared to the metrics involving signal phase^33^. Therefore, in this study we focus on this type of coupling. Power-to-power coupling was calculated for the ‘good’ ICs for each EEG segment. The resulting CFC matrices were calculated between all frequencies 2–120 Hz pairwise (logarithmic scale) using a modified script based on the PowPowCAT toolbox for EEGLAB^34^.

The CFC matrices (averaged over the ‘good’ ICs for each segment) were used as training and testing sets for the Stacked Sparse Autoencoder (SSAE). This machine learning method begins by using unsupervised training to find the most characteristic features of the input classes and thus reduces the dimensionality of the inputs. This feature may be important to make the data analysis more robust against the intrinsic noise and individual variations of the EEG signal. The SSAE network was created with MATLAB (v. R2021b) and consisted of two encoder-decoder networks and the softmax layer with two outputs for binary classification ‘seizure vs. background’. Half of the EEG segments were selected randomly for network training and the remaining half were used for testing.

## Results

Among the 12 patients whose data were used in this study, there were 5 males and 7 females. The max/min age of the patients was 22/4 years and the average age was 10±6.1 years. Demographic data of patients and clinical characteristics of their absence seizures are presented in Table 1. Half of patients had ‘atypical’ absence seizures due to their ‘focal’ features at the onset (for example, seizure activity predominantly at the frontal or temporal electrodes with rapid secondary generalization) or the presence of minor muscular phenomena (eye blinking or involuntary twitching).

**Figure 1:**
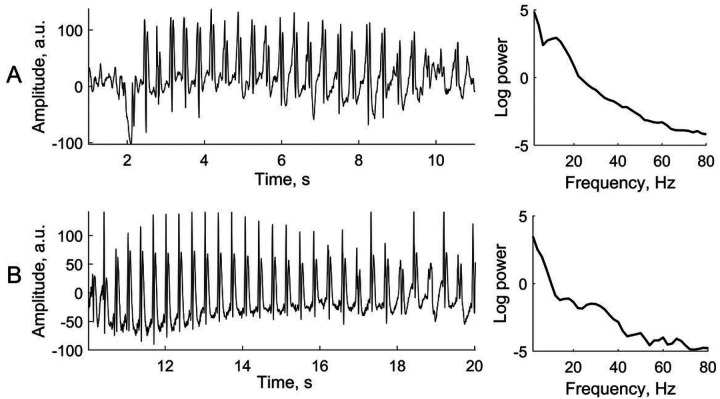
Representative examples of EEG activity during absence seizures from two patients. *Left,* EEG traces showing typical waveforms of the 3-per-second spike-and-wave rhythm. *Right,* the corresponding power spectra of the segments shown on the left (linear frequency scale).

Two typical examples of EEG activity during absence seizures from two patients are presented in Fig 1, *left* along with the corresponding power spectra (Fig 1, *right*). The EEG activity showed a typical spike-wave pattern with the repetitive rate of around 3 cycles per second. Quite often, the spike-and-wave cycle contained not a single but multiple spikes preceding the slow wave and this resulted in a relatively broad spectral peak in the theta-alpha range 3–12 Hz (Fig 1, *right*). Not infrequently, the spectrum of the spike-and-wave rhythm would contain higher frequencies in the beta-gamma range 20–80 Hz (Fig 1B, *right*).

**Figure 2:**
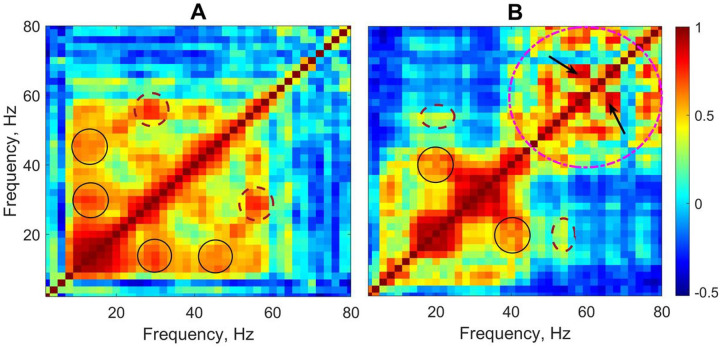
Cross-frequency matrices of EEG segments shown in Fig 1, A and B, respectively (linear frequency scale). Circles, ovals and arrows show examples of a relatively stronger coupling between different frequencies including both harmonic and non-harmonic relations. See text for details.

The corresponding cross-frequency matrices for EEG segments shown in Fig 1 are presented in Fig 2 where panels A and B correspond to the EEG segments shown in Fig 1A and Fig 1B, respectively. The overall pattern of the power-to-power frequency coupling was characterized by multiple discrete local maxima forming a ‘grid’ always symmetrical along the main diagonal. An approximately equal spacing between those maxima suggested that they reflected cross-frequency coupling between *harmonics*. Harmonics are integer multiples of the fundamental frequency arising in the spectral domain as a consequence of the main waveform not being strictly sinusoidal. Therefore, a relatively high coupling between the main frequency and its spectral harmonics is expected because harmonics occur at predictable intervals within the main waveform. For example, in Fig 2A some maxima (off the main diagonal) are present at the xy-coordinates of (15, 30) & (30, 15) Hz and (15, 45) & (45, 15) Hz (black solid circles). These maxima are likely to represent harmonics of the main frequency 15 Hz. Also, there are maxima at (28, 56) & (56, 28) Hz (brown dashed circles) which represent the first harmonic of frequency 28 Hz. Similarly, in Fig 2B there are maxima at the xy-coordinates of (20, 40) & (40, 20) Hz (black solid circles) which represent the first harmonic of the main frequency 20 Hz.

The cross-frequency patterns in the data, however, were not limited to the harmonics of the frequencies within the beta range. For example, figure 1B also shows maxima at the coordinates of (20, 54) & (54, 20) Hz (the brown dashed ovals), and clearly the frequencies 20 Hz and 54 Hz are *not* harmonically related. Moreover, there are multiple maxima within the gamma band 40–80 Hz (the pink dashed circle) which demonstrate the coupling of gamma frequencies not harmonically related to each other (e.g., 58 and 66 Hz, arrows in Fig 2B).

**Figure 3:**
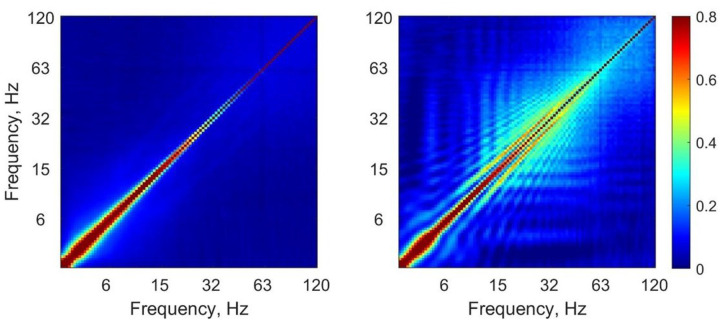
Cross-frequency coupling matrices group-averaged over all background segments (*left*) as well as absence seizures (*right*) (logarithmic frequency scale).

Cross-frequency coupling matrices group-averaged over all background as well as absence seizure EEG segments are shown in Fig 3. The matrices demonstrate that the major difference between background and seizure activity was due to an increase in the power-to-power coupling within the beta-gamma bands (from about 15 to 70–80 Hz) during seizures.

**Figure 4:**
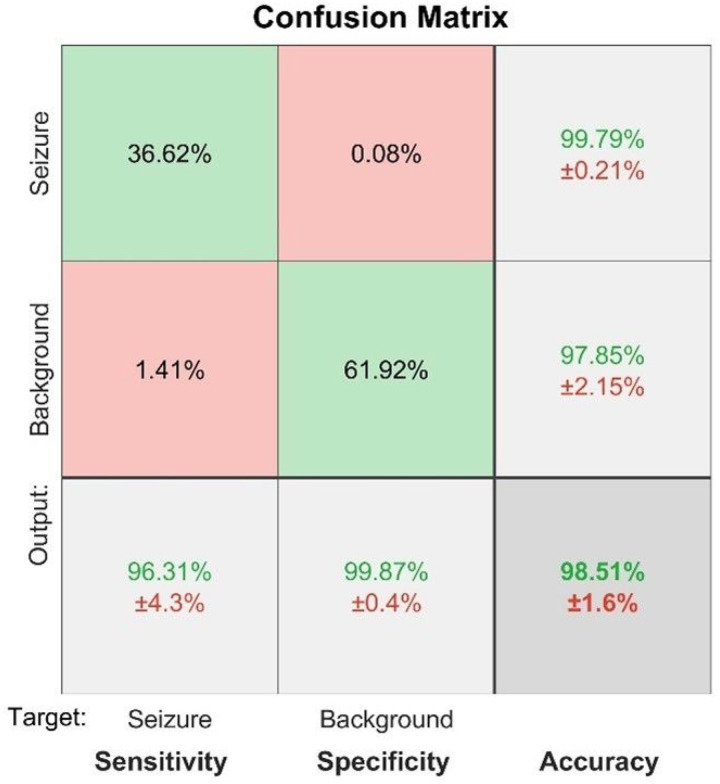
Confusion matrix showing the results of recognition of seizures and background segments by the SSAE network. The mean values (%) ± standard deviations (%) are shown for sensitivity, specificity and overall accuracy (the bottom row) as well as for positive predictive values for each class (two upper cells in the far right column).

During training, the network with L2 and sparsity regularizers achieved a squared error smaller than 10^−2^ with about 400 iterations. Figure 4 shows the confusion matrix with the results of recognition of seizures and background segments by the network. The trained network was able to correctly classify EEG segments (not used in training) at a sensitivity of 96.3%, a specificity of 99.8%, and an overall accuracy of 98.5%. A false positive rate of 0.08% (Fig 4) translates to about ~3 false alarms per hour.

## Discussion

The use of machine learning (including deep learning neural networks) in studies attempting to recognize and predict absence seizure EEG activity has been rapidly advancing in the past decades, generating promise in improving both clinical treatment as well as the neurobiological understanding of this disorder. Studies since the early 90’s describe the ability of machine learning to recognize absence seizures with high level of sensitivity (~95%) albeit often with higher rates of false positives^35,36^. Many of these earlier studies used genetic murine models of absence epilepsy and implanted EEG electrodes. More recent ones apply these techniques to humans using only scalp EEG and with the ability to run the computation not only offline, but also in real time.

In the last decade, machine learning in murine models have overturned the notion of absence seizures as unpredictable and spontaneous by showing success beyond recognition to prediction of such seizures^37^. This ability to differentiate the pre-seizure from the seizure state is now being successfully applied to humans using only scalp EEG with as few as 19 scalp electrodes^38,39^. The murine models employed in studies on absence epilepsy exhibit notable high sensitivity and specificity, often surpassing 95% for accurate recognition. Luijtelaar et al. achieved a good sensitivity of up to 90% with a low rate of false positives^37^. Li et al. demonstrated an impressive accuracy of 97.5% in distinguishing between background, preictal and ictal states in human EEG^38^. In a more recent exploration with a shallow convolutional neural network applied to scalp EEG data from human subjects, Zhang et al. achieved a sensitivity of 92.2% (not limited to absence-seizures) with a low rate of 0.12 for false positives^40^.

Ongoing research in machine learning is actively exploring absence seizures to identify their critical features, aiming to gain deeper insights into the electrophysiological roles that these features play, with the goal of improving seizure prediction. In both murine and human studies, successful training of the networks usually involves using relevant time and frequency domain metrics especially frequency and amplitude, and sometimes phase^39,41–43^. Most studies use wavelet analysis techniques to account for non-stationarity of the EEG signal and improve time and frequency localization of various EEG patterns. Entropy-related metrics, especially permutation entropy (PE), was also very useful in training networks, and decreases in PE were found in both preictal and ictal segments in comparison to background^38,44^. Furthermore, specific spatial features were found to characterize absence seizures such as increased cortico-thalamo-cortical synchrony in murine models, or reductions in overall functional connectivity patterns during generalized spike-and-wave discharges in humans^37,39^.

Many studies found that the harmonics of the fundamental frequencies of seizures are highly specific and critical to the machine learning success^45,46^. Harmonic spectral analysis involves broad wavebands (i.e. 1–100Hz) that include HFOs which are increasingly recognized as crucially important in the pathophysiology of epilepsy. The energy in these higher frequency harmonics are found to be important signatures differentiating between regular sleep spindles, artifacts and true spike-and-wave discharges that all share the same fundamental frequency^45^. The interdependent and harmonic architecture of the EEG frequency spectrum has been well described by authors such as Buzsáki^47^ and Klimesch^48^ and indicates that a comprehensive analysis of EEG activity should involve a view of the cross-frequency dynamics. It is noteworthy that incorporating knowledge of cross-frequency coupling patterns holds promise for further enhancing the optimization between sensitivity and specificity. This becomes particularly crucial in scenarios where data is less pristine or encompassing multiple states such as sleep and wakefulness. It is becoming clear that there is a complex interplay between spectral, harmonic and spatial features that can reliably characterize absence epilepsy. Cross-frequency analysis is a powerful tool to look for novel biomarkers of absence seizures. Similarly, such analysis would deepen our knowledge of the underpinnings of absence seizures by further clarifying the involvement of HFOs (which are already known to be deeply related to seizures), harmonic patterns, as well as interdependent relationships between different frequency bands more generally.

## Conclusion

Our results provide evidence that the parameters of cross-frequency coupling and the SSAE neural networks can be used for automated detection of seizures within scalp EEG. Importantly, the trained SSAE network showed generalizability in detecting seizures with sensitivity, specificity and accuracy higher than 96% with all patients tested. Automated analysis based on deep learning networks can significantly accelerate the analysis of EEG data and increase their diagnostic value.

## Figures and Tables

**Table 1.** Demographic information and clinical features of patients’ absence seizures

Subject #	Gender	Age	Clinical features of absence seizures
675	F	4, 6	Atypical absence seizure (blinking).
1113	F	20	Absence seizures.
1413	F	10, 14	3 to 6 Hz generalized spike and slow wave activity; seizures lasting 10–16 seconds.
1795	F	9	Atypical absence seizures. 3 to 5 Hz spike and slow wave activity preceded by symmetric focal (frontal) activity.
1984	M	6	Atypical absence associated with involuntary twitching and motion arrest.
2448	M	4	Typical of absence seizures but with a possibility of a secondarily generalized mechanism including the left frontal activity seen at the onset.
2657	M	5	Multiple absence seizures.
3053	F	5	EEG suggests more than one mechanism for seizures in this patient.
3281	M	13	The seizures were frontally predominant and relatively characteristic of absence epilepsy.
3306	F	13	Typical absence seizures.
3635	M	6	Generalized SW discharge with a clear underlying frontal focality.
8608	F	22	Atypical absence seizure with focal features (subtle focal slowing and sharp waves at T3, T5 and C3).

## Data Availability

The data of the secondary analysis in this study are available on request from the corresponding author (A.V.M.). The original EEG data are publicly available from the Temple University Hospital database (the TUSZ corpus,^31^) (https://isip.piconepress.com/projects/tuh_eeg).
